# How Stress Treatments Influence the Performance of Biodegradable Poly(Butylene Succinate)-Based Copolymers with Thioether Linkages for Food Packaging Applications

**DOI:** 10.3390/ma10091009

**Published:** 2017-08-30

**Authors:** Valentina Siracusa, Laura Genovese, Andrea Munari, Nadia Lotti

**Affiliations:** 1 Department of Chemical Science, University of Catania, Viale A. Doria 6, 95125 Catania (CT), Italy; 2 Dipartimento di Ingegneria Civile, Chimica, Ambientale e dei Materiali, Università di Bologna, Via Terracini 28, 40131 Bologna, Italy; Laura.genovese@unibo.it (L.G.); andrea.munari@unibo.it (A.M.); nadia.lotti@unibo.it (N.L.)

**Keywords:** poly(butylene succinate)-based copolymers, biodegradable polymers, gas barrier properties, polymer aging, food simulant

## Abstract

Biodegradable poly(butylene succinate) (PBS)-based random copolymers containing thioether linkages (P(BSxTDGSy)) of various compositions have been investigated and characterized from the gas barrier, thermal, and mechanical point of view, after food contact simulants or thermal and photoaging processes. Each stress treatment was performed on thin films and the results obtained have been compared to the same untreated film, used as a standard. Barrier properties with different gases (O_2_ and CO_2_) were evaluated, showing that the polymer chemical composition strongly influenced the permeability behavior. The relationships between the diffusion coefficients (*D*) and solubility (*S*) with polymer composition were also investigated. The results highlighted a correlation between polymer chemical structure and treatment. Gas transmission rate (*GTR*) mainly depending on the performed treatment, as *GTR* increased with the increase of TDGS co-unit amount. Thermal and mechanical tests allowed for the recording of variations in the degree of crystallinity and in the tensile properties. An increase in the crystallinity degree was recorded after contact with simulant liquids and aging treatments, together with a molecular weight decrease, a slight enhancement of the elastic modulus and a decrement of the elongation at break, proportional to the TDGS co-unit content.

## 1. Introduction

Since the last decade of the 20th century, packaging has been among the fastest growing areas of plastics. The remarkable success of these materials is due to a combination of interesting characteristics leading to excellent performance. Light weight, flexibility, strength, transparency, and ease of sterilization are some of the property requirements for polymers used in packaging applications. By combining the abovementioned properties in different ways, it is possible to obtain plastics useful for different applications, ranging from flexible films to rigid containers. Unfortunately, packaging materials are often used for short shelf life applications and in most cases recycling is not sustainable and economically convenient owing to the contamination with organic matter, especially when used in the food sector [1]. This massive consumption is accompanied by a consistent waste generation that causes important waste management issues. Indeed, as reported by Plastic Europe [2], more than 30% of landfill is plastic waste.

Thus, the growing concern about environmental pollution, the exploitation of finite fossil fuel resources, and waste management issues are the driving force for governments, companies, and scientists from all over the word focusing on finding a suitable alternative to petroleum-based traditional polymers [3,4,5,6,7,8]. As reported by Reddy et al. [9], overdependence on petroleum resources can be relieved by the use of bioplastics.

Bioplastics, i.e., plastics obtained from renewable resources and/or biodegradable, may represent a solution to these urgent needs. As reported by European Bioplastics [10], their actual market is 4.1 Mtons/year and the growth expectation is about 50% in five years, at a much higher rate with respect to traditional fossil-fuel-derived plastics [6].

Bioplastics can be obtained from 100% renewable materials, from biodegradable fossil-based polymers, or a combination of both. However, with respect to petroleum-derived plastics, bioplastics present different disadvantages such as lower performance and processing and higher costs. Furthermore, poor gas barrier properties, unbalanced mechanical properties, and low melting temperatures have until now limited their use for packaging applications. Copolymerization could help in improving the properties discussed above. In fact, it is well known [11,12,13] that copolymerization could be used as a tool to overcome these limitations by obtaining novel materials with a broad range of chemical–physical, mechanical, and gas barrier properties that can be easily modulated by varying the chemical nature of the co-units and their distribution in the molecular backbone.

Within the broad class of bioplastics, aliphatic polyesters represent good examples of biodegradable bioplastics, being compostable and characterized by good mechanical and gas barrier properties at competitive cost [14,15,16,17]. Among these, poly(butylene succinate) (PBS) is a biodegradable high molecular weight polyester obtained by direct polymerization of succinic acid and 1,4-butanediol. Currently, as reported by several researches [9,18,19], great efforts are being made in order to obtain succinic acid and 1,4-butanediol from biological feedstocks such as corn starch, corn steep liquor, whey, and cane molasses, or from bacteria [20,21]. However, PBS alone has a high degree of crystallinity and rigidity that limits its use in applications where rapid degradation rate and higher flexibility are required. As reported above, copolymerization allows us to overcome these limits [11]. From this view, in the past, ether and thioether linkages have been introduced along with a PBS polymer backbone. Our previous research work [14,22,23] found that these modifications indeed speed up hydrolytic and enzymatic degradation, mainly due to a lowering of the degree of crystallinity and an increase in surface film hydrophilicity, and increased chain flexibility.

Taking into account all these considerations, we studied a new class of PBS-based random copolymers containing thioether linkages, previously synthesized by some of us [11]. Their chemical–physical, mechanical, compostability, and basic barrier behavior have been fully investigated [11] and correlated to their chemical composition. In order to consider these materials in food packaging applications, deeper analyses have been performed in this work. Considering that plastics must not affect the taste, quality, or shelf life of the food [24], severe control of the gas barrier property is required. This, in turn, is dependent on the structure of the polymer packaging material. Consequently, gas barrier behavior becomes one of the most important characteristics of materials used for food packaging applications, because food shelf life and storage conditions are strongly dependent on their performances at different temperatures and in the presence of different gases and moisture contents. Gas permeability is linked to several polymer properties that can be modulated by tailoring the polymer chemical structures. A detailed characterization of these materials is consequently of fundamental importance to verify their suitability for a specific application.

Since up to now barrier properties under stress conditions on PBS-based copolymers were never studied, in this study, the effect of thermal and photoaging as well as of contact with food simulant fluids on mechanical and permeability properties has been evaluated on poly(butylene succinate)-based copolymers containing thioether linkages. The properties of these new materials have been proven to be strictly related to the chemical composition and linked to their molecular and thermal properties.

## 2. Results and Discussion

**Scheme 1:**
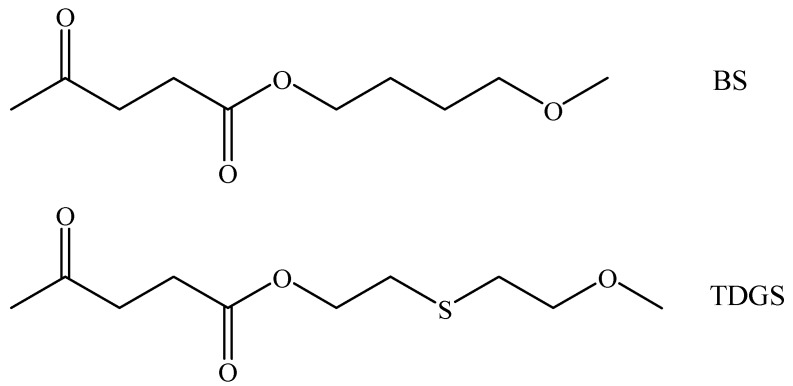
Chemical formulas of BS (**above**) and TDGS (**below**) comonomeric units.

In Scheme 1 the chemical formulas of the monomeric units of the two homopolymers are reported.

The two comonomeric units are very similar, with a saturated aliphatic chain containing two ester groups. As can be observed, they only differ by the presence of an additional sulfur atom in the TDGS unit.

All the synthesized polymers appear at room temperature as light yellow semicrystalline materials. They present high and comparable molecular weights, confirming good polymerization control as well as appropriate synthesis conditions. On Table 1 are reported the characterization data for the neat polymers.

As previously reported [11], water contact angle analysis (WCA) evidenced a more hydrophobic behavior of PBS with respect to PTDGS, as the introduction of a sulfur atom per repeating unit increases the material hydrophilicity. As a consequence, when increasing the TDGS co-unit content, a decrease in WCA has been recorded. Furthermore, although the thermal stability was good for all copolymers, it regularly decreased with the increase in TDGS co-units because of the lower energy associated with the C–S bonds with respect to the C–C ones. In addition, by increasing the TDGS unit content, both the melting temperature and the heat of fusion decreased, and the presence of a larger distribution of the less perfect and smaller crystallites, also confirmed by X-ray diffraction (XRD) (X’Pert PANalytical diffractometer, Lelyweg 1, 7602 EA, Almelo, The Netherlands), was highlighted. The copolymer composition did not influence the glass transition phenomenon as the T_g_s of the copolymers was similar and well below the room temperature (−34 to −38 °C), revealing a soft amorphous phase. Lastly, as regards the mechanical properties, a decrease in the elastic modulus with an increase in the number of TDGS co-units, and an opposite trend for the elongation at break, was found. These results have been explained on the basis of the lower crystallinity degree and the higher chain flexibility, due to the presence of longer C–S bonds with respect to C–C ones.

After the treatments, the polymers were subjected to molecular, thermal, mechanical, and barrier properties evaluation. The results have been compared to those of untreated samples to determine the effect of contact with the simulant liquids and of the stressed aging process on the polymer performances.

### 2.1. Molecular Weight Determination

The molecular weight of the polymers after the contact with food simulants was measured and the results are reported in Table 2, as % of residual M_n_.

The treatment with simulant D did not influence the polymers’ molecular weight, while an appreciable degradation was observed when in contact with the other liquids. Greater effects have been observed when the film samples were in contact with simulant C. The observed trend can be explained considering both the degree of crystallinity and the polymer surface wettability. As reported in the literature, the lower the crystallinity degree and the surface hydrophobicity, the higher the degradation rate [23,25,26]. With simulant C, the hydrolitic degradation was accelerated by the acid solution, with higher molecular weight degradation compared to the other A and B simulants.

In Table 3, the residual M_n_ (%) after thermal and photoaging processes is reported.

The thermal treatment caused a decrease in molecular weight in all polymers under investigation. PBS homopolymers lost about 18% of the initial M_n_, while the copolymers degraded to a higher extent. In particular, we observed a decrease of 18% for P(BS80TDGS20) and of 27% for P(BS60TDGS40). The photoaging process produced an even greater effect on all samples. In particular, a decrement of 37% was observed for PBS homopolymer, 40% for P(BS80TDGS20), and 100% for P(BS60TDGS40). The higher the amount of TDGS co-units, the higher the degradation rate—up to P(BS60TDGS40), whose decomposition was complete. The results could be explained by the presence of a higher amount of lower energy C–S bonds versus C–C ones. Despite the concentration of the ester groups being similar in all the samples, the presence of an increasing number of sulfur atoms, together with a lower degree of crystallinity, promoted the degradation of the polymer chains [14,27,28]. This behavior was far more evident during photoaging than thermal aging.

### 2.2. Thermal Properties

A calorimetric study was performed on the samples after contact with food simulants and after thermal and photoaging. The data obtained are reported in Table 4 and Table 5, respectively, as % of ΔH_m_, associated with the residual crystallinity.

After food simulant contact, different thermal behavior was recorded for all samples under investigation. From the data reported in Table 4, the ΔH_m_, associated with the percent of crystallinity, showed some changes. For PBS it was more evident after simulant D1 contact, with a decrement of 36%: this conspicuous decrement influenced the gas barrier permeability. In fact, the permeability-detected value was the highest, in agreement with the well-known interdependence of crystallinity and the gas permeation process [29]. For P(BS80TDGS20), no appreciable changes were observed; a negligible difference was observed in the T_m_ values (not reported), indicating that the molecular weight of the materials does not change significantly after food contact with the simulant liquids. For P(BS60TDGS40) a different behavior was recorded, depending on the treatment applied. For this sample, during the food contact treatment a decrease of the highest temperature peak was recorded, accompanied by a broadening of the endothermic peak. This thermal behavior can be associated with the chain scission processes of long polymer chains, resulting in the formation of shorter ones able to crystallize easy.

As far as thermo-aging effects are concerned, the highest ΔH_m_ reduction was recorded for the P(BS60TDGS40) sample, about 29%. As previously indicated, the higher the TDGS co-unit content, the lower the resulting stability. On the contrary, no appreciable effect of time exposure was found.

In the case of photoaging, different behavior was observed. First of all, the samples under investigation behaved in a different way. In some of them, an increase of heat of fusion was observed as a consequence of a higher crystallizing capacity of the shorter polymer chains created by the chain scission process; in other cases, the degradation process occurred to such an extent that the polymer chains became too short to be able to chain fold and therefore crystallize. Also in this case, the worst behavior was observed for the sample with the highest TDGS co-unit content P(BS60TDGS40), which ended up completely amorphous. Similar behavior was recorded by Siracusa et al. [30] on poly(butylene succinate) and poly (butylene succinate-co-adipate) commercial samples, used for packaging applications, after stress treatments and was observed by Gauthier et al. [31] during their study on the photo- and thermal degradation of polyethylene-based films.

### 2.3. Mechanical Characterization

For future application as a packaging material, the study of the mechanical properties is of great importance. Therefore, the P(BSxTDGSy) copolymers were subject to stress–strain measurements after stress treatments in order to investigate any possible modification of their mechanical behavior. Tensile tests were performed on the films.

In Table 6 are reported the mechanical characterization data after food simulant contact, while in Table 7 and Table 8 are reported the data obtained after thermal and photoaging.

The mechanical properties of the untreated samples were found to be well correlated with the chemical composition of the polymer samples, as well as in their degree of crystallinity. As is reported in the literature [32,33], the degree of crystallinity has an important effect on the mechanical performance of the polymers. In particular, higher crystallinity leads to harder, stiffer, and less ductile behavior, implying higher elastic modulus (E) and lower elongation at break (ε). As expected, for untreated samples, by increasing the TDGS co-unit content, the elastic modulus decreases and the elongation at break (ε_b_) increases, due to the decrease in crystallinity degree and the increase in chain flexibility. The P(BS60TDGS40) sample showed the lowest elastic modulus (160 MPa) and the highest elongation at break (810%). After contact with the food simulant liquid, a general modification of the modulus and elongation at break was observed, probably due to the degradation induced by the treatment. In the case of PBS and P(PS80TDGS20) samples, a general trend of the mechanical behavior was observed, as evidenced by a decrease in both the elastic modulus and elongation at break. For sample P(BS60TDGS40) different behavior was found, well correlated with the molecular weight and thermal data. For this sample, the molecular weight was not remarkably affected by the contact with simulant liquids and, moreover, a high increment of the crystallinity was measured. As a matter of fact, the corresponding elastic modulus increased, while the elongation at break decreased. Despite the higher crystallinity of such a sample, a high value of elongation at break was recorded. The observed trend can be ascribed to the presence of longer C–S bonds with respect to the C–C ones, which confers chain flexibility to the copolymer macromolecular chains.

After aging treatments (at 10 or 20 days and at the end of the experiments), a general decrease in the elastic modulus as well as in the elongation at break was observed for all samples, though to a different extent. Such changes were more pronounced for the photoaged samples than for the thermal ones, in agreement with molecular weight and thermal variation data, which proved that the photoaging process had a stronger effect than thermal aging. The P(BS60TDGS40) sample suffered the largest worsening of the mechanical properties, as the film broke easily during handling, underlining a significant degradation.

### 2.4. Barrier Properties

As can be observed from the chemical formulas, the comonomeric unit introduced along PBS polymeric chains differs from a sulfur atom with respect to the BS polymer sequence. Considering that the gas barrier behavior depends on the specific molecular structure of the polymer involved, for food packaging applications knowing the permeability behavior is very important, especially when gases are used as a tool to improve the food’s shelf life. Therefore, gas permeability studies are critical to understanding barrier materials’ behavior in order to estimate their utility [34]. Concerning the theory, gas permeation through a polymer is described by a diffusion model described by Henry and Fick’s laws, which allow us to obtain the expression that relates the permeation rate to the area and thickness of the film. The permeability behavior is therefore expressed as the Transmission Rate (TR) of the material: TR = Q/A*t* where Q is the amount of permeant passing through the film (*cm*^3^), A is the sample area (*cm*^2^), and *t* is the time (*day*). Barrier gas properties of polymers to gases or water are often presented in this way, and the term *GTR* (Gas Transmission Rate) is in common use [35,36].

*GTR* standard values, together with *S*, *D*, and *t_L_* ones, of the tested gases were reported previously by Genovese et al. [11]: a good correlation with the chemical copolymer composition was found, with the possibility of tailoring the barrier behavior with respect to the desired application. In particular, the authors observed a decrease in the barrier performance (higher *GTR* value) with the increase in % TDGS unit content, due to a decrease of crystallinity percentage. This behavior was more evident with CO_2_ gas test than O_2_, in agreement with the literature data [36].

*GTR*, *S*, *D*, and *t_L_* parameters for the stressed samples, after simulant liquid contact and during thermal and photo exposure of the samples, were evaluated in order to make a correlation between the gas transmission behavior and the main influencing factors.

#### 2.4.1. Simulant Liquids

**Figure 1:**
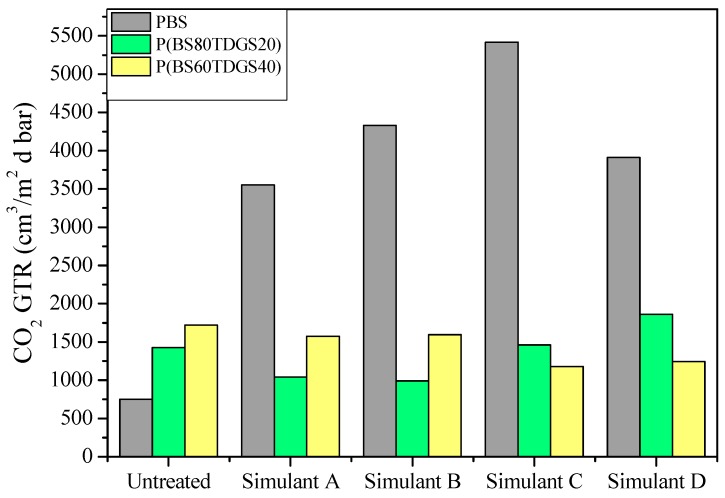
CO_2_ *GTR* after food simulant contact for PBS and P(BSxTDGSy) copolymers.

In Figure 1, as an example, GRT data recorded after food simulant contact, with CO_2_ gas testing, of the untreated and treated samples are reported. In Table 9, the *GTR*, *S*, *D*, and *t_L_* values of the CO_2_ gas test, with the corresponding increment (>) or decrement (<) with respect to the untreated sample (in brackets) are collected. In Table 10, the corresponding permselectivity ratios obtained for the untreated samples, from [11], are indicated for the sake of simplicity.

As reported in the literature [34,37,38], the term permselectivity is correlated to the permeation ratio between gases. In general, as reported by Muller et al. [39], the ratio O_2_:CO_2_ for many polymers is in the range of 1:4 and could be used to calculate the permeability of one gas by knowing the permeability of the other one. However, this parameter is correlated with the chemical composition of the polymers under investigation and so a general trend could not be obtained [34]. In fact, as Table 10 shows, the values change with the copolymer composition.

For the untreated samples, the different chemical structure, i.e., copolymer composition, played a key role in determining the gas barrier behavior. This last was explained on the basis of crystallinity degree and polymer molecular weight. As is well known from the literature [40,41], highly crystalline samples showed low permeability but could also have the reverse effect. In fact, in some cases, highly crystalline samples present higher gas permeability due to the de-densification phenomenon of the amorphous phase caused by the presence of small areas of the crystalline phase. In our study, we found that the presence of C–S bonds facilitates the CO_2_ gas crossing through the polymer membrane (see Table 10). From Figure 1, it can be observed that the untreated sample increases their CO_2_-GTR value by increasing the % of the TDGS co-unit. The treated samples show different behavior. In fact, PBS permeability performance is significantly worse when in contact with the simulant liquid, especially with Simulant C, despite its high hydrophobicity. According to the literature [42], under the action of water the polymer swells, making the diffusion of the gas molecules through the film easier. Concerning P(BSxTDGSy) copolymers, different behaviors were recorded. The copolymer with the highest content of TDGS co-units even shows an improvement of CO_2_ GTR gas barrier performance, while the other samples showed a general worsening of the barrier behavior. The increase in chain flexibility, due to the presence of C–S bonds, balanced the swelling of the copolymers under the effect of the simulant liquid. This was supported by the *D*, *S*, and *t_L_* data. The *S* value expresses the volume solubility of the gas dissolved in one volume of polymer material. The *D* value is correlated with the gas mobility inside the polymer membrane, while *t_L_* is correlated to the time requested to reach the steady-state of the permeation process. These data are influenced by the chemical structure of the polymers. Following the theoretical behavior, if the gas transmission increases an enhancement of the solubility is recorded, with a consequent diffusion decrease [14]. As an example, from Table 10, for the PBS sample the *S* value with CO_2_ is about 272 times higher than O_2_, indicating a higher compatibility of this gas with the polymer than O_2_. Consequently, the *D* value is lower with CO_2_ because this gas spent more time crossing the film and thus the time to attain the steady-state is longer because the molecules need more time to homogeneously arrange inside the polymer membrane. After treatment with simulant liquids, due to the higher decrement of crystallinity, the gas transmission through the films increased, balanced by the increased chain mobility. A theoretical trend could not be confirmed.

#### 2.4.2. Thermal Aging and Photoaging

**Figure 2:**
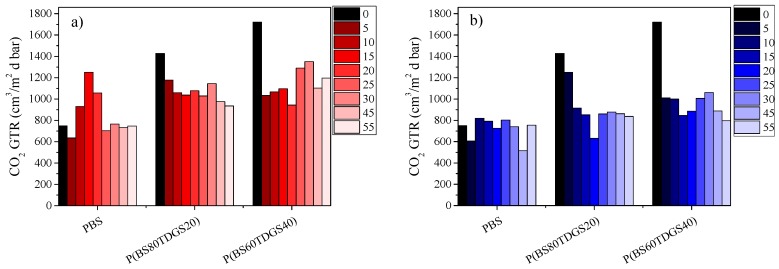
*GTR* data for PBS and P(BSxTDGSy) copolymers, after: (**a**) thermal aging (at 40 °C, 50% RH, air ventilated); (**b**) photoaging at 23 °C, D65 Neon light, 50% RH, air ventilated.

Thermal and photoaging were conducted in order to accelerate the degradation process and simulate a supermarket exposure, respectively. The thermal treatment performed corresponds to an aging of 0.7–8.3 solar years (365 days after the first exposure, so 255.5–3029.5 days of aging), calculated according to the literature [43,44]. Data recorded are reported in Figure 2 with the corresponding linear regression coefficients (R^2^) of the corrected experimental point reported in Table 11.

The *GTR* trend after thermal and photoaging was explained taking into consideration several factors. As is well known [40], an increment of the crystallinity percentage causes a decrement in the *GTR*. However, it has to be remembered that both stress treatments could promote polymer inter-chain interactions, which are favored in the samples under investigation by the glycerol (present in the same quantity in all the polymers) and by the polarity of the *S* atoms.

PBS behaves differently according to time exposure: for a short exposure, a worsening of the gas barrier behavior was observed, associated with a decrement of crystallinity (lower ΔH_m_) and molecular weight; for a long exposure, a decrement in the *GTR* was observed, with the value becoming similar to that of the untreated sample thanks to polymer inter-chain interactions, promoted by the thermal treatment, whose effect is greater than that correlated with the decrement of crystallinity. The two copolymers were characterized by similar behavior: interestingly, barrier performance in both cases improved significantly, due to both the increment in the crystallinity degree and to inter-chain interactions, favored by the presence of sulfur atoms along the polymer chains. In fact, it is worth noting that P(BS60TDGS40) showed the best improvement, in line with the highest amount of S atoms. As to the photoaging, all samples under study showed similar behavior: a linear decrease in the *GTR* with elapsing time was indeed observed. At day 55, the end of the experiment, similar barrier properties had been achieved for all the samples under investigation, despite the different barrier performance of the untreated polymers. The results obtained could be explained on the basis of the intense inter-chain interactions, which are favored by light and by increasing the sulfur content. The different behavior found for PBS homopolymers, depending on the different stress treatment applied, can be explained by taking into account that polymer inter-chain interactions are mainly promoted by light, as previously observed for other polymer samples [14,30].

Concerning the *S* and *D* values, as described in the literature [35], they are parameters correlated to the gas–polymer interaction observed during the gas barrier study. They follow four assumptions only in an ideal case: (i) they are independent of the gas concentration; (ii) the diffusion process occurs under steady-state conditions; (iii) the gas–concentration relationship through the polymer is linear; and (iv) the diffusion process takes place only in one direction.

When the materials under study are subjected to stress treatments, considerable interaction between the polymer and the permeants takes place, overruling the theoretical behavior.

**Figure 3:**
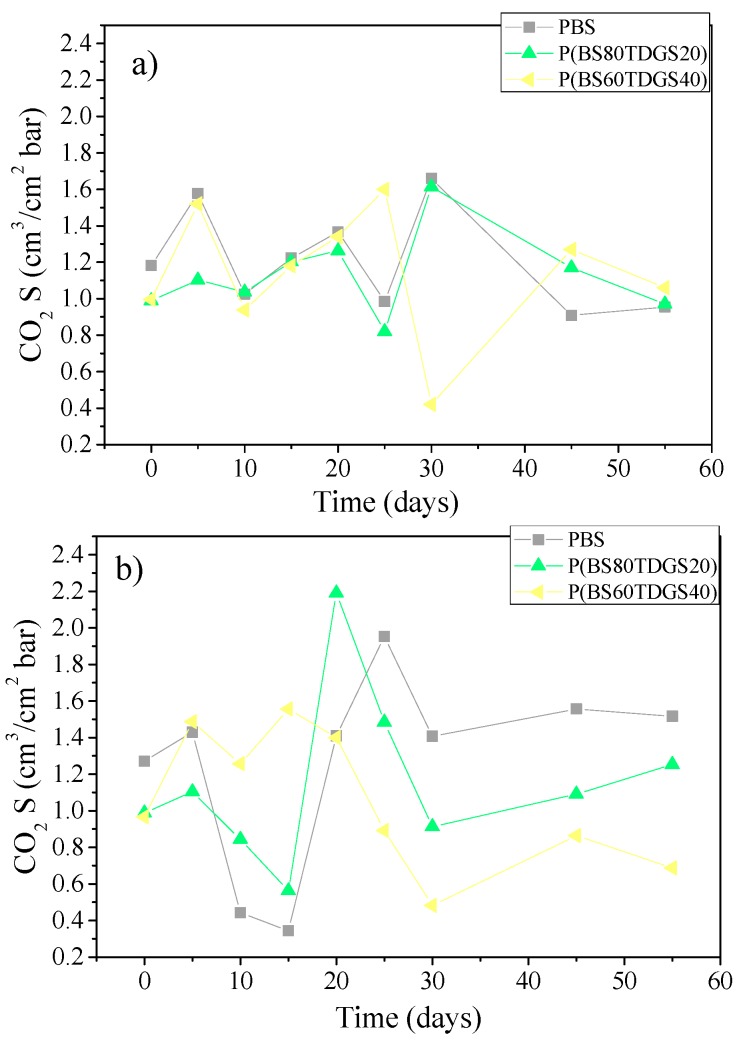
*S* coefficients for PBS and P(BSxTDGSy) copolymers after: (**a**) thermal aging (at 40 °C, 50% RH, air ventilated); (**b**) photoaging at 23 °C, D65 Neon light, 50% RH, air ventilated.

**Figure 4:**
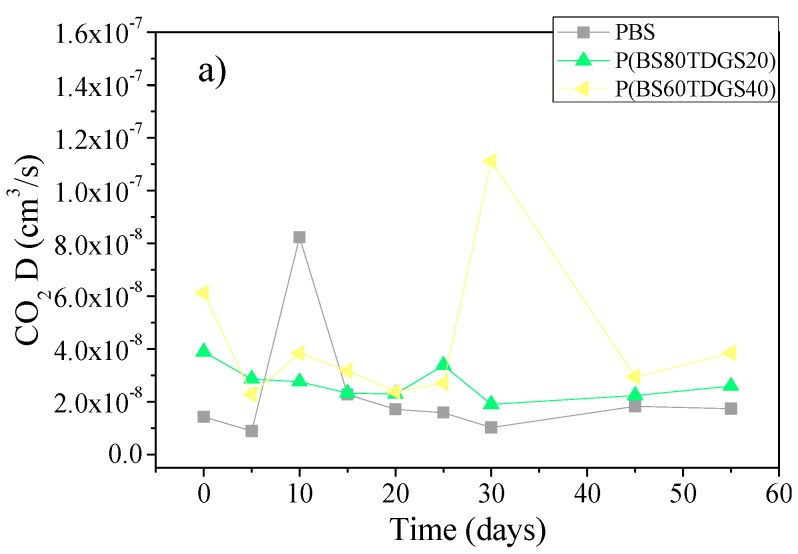

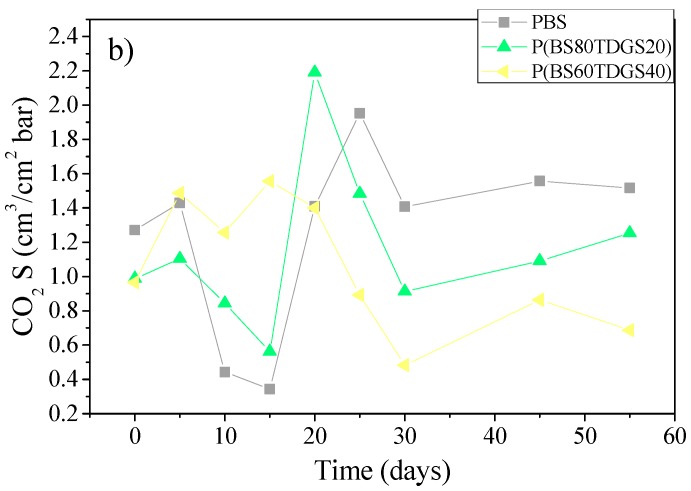
*D* coefficients for PBS and P(BSxTDGSy) copolymers after: (**a**) thermal ageing (at 40 °C, 50% RH, air ventilated); (**b**) photo-ageing at 23 °C, D65 Neon light, 50% RH, air ventilated.

**Figure 5:**
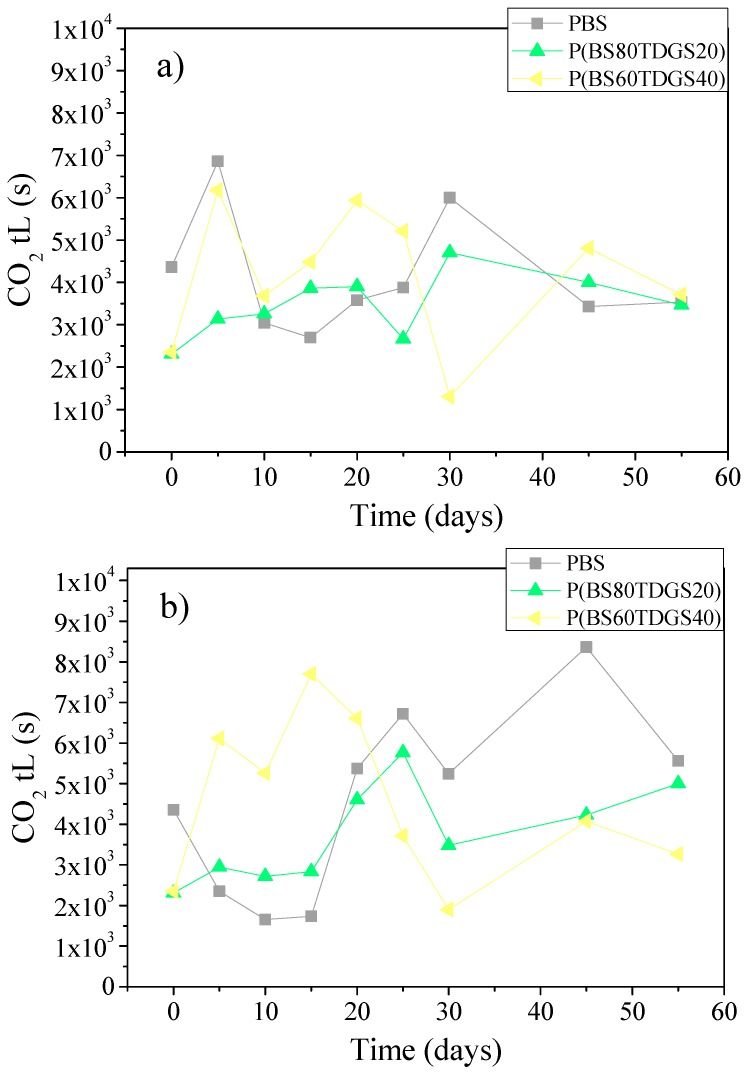
*t_L_* coefficients for PBS and P(BSxTDGSy) copolymers after: (**a**) thermal ageing (at 40 °C, 50% RH, air ventilated); (**b**) photo-ageing at 23 °C, D65 Neon light, 50% RH, air ventilated.

In Figure 3, Figure 4 and Figure 5 are reported the *S*, *D*, and *t_L_* data recorded after thermal and photoaging, respectively.

Although the steady-state of the permeation process is normally achieved in a few hours, with larger molecules such as CO_2_ reaching the steady-state could take a longer time, promoting the interaction between polymer and permeates. It is for this reason that the fluctuation of the permeation coefficients could be recorded. In fact, S and D data show different behavior than the theoretical. In general, an increase of S corresponds to a decrease of D and vice versa. We observed a fluctuating value, especially within the 30 days and under thermal aging treatment. Starting after 35 days of exposure, a soft equilibrium was reached, more intense under photoaging.

### 2.5. FT-IR Spectroscopic Data

FT-IR spectra (Perkin-Elmer-1725-X Spectrophotometer, Labexchange Group, Burlandingen, Germany) were recorded for each sample in order to investigate the change in the chemical structure due to the stress treatments. The principal absorption bands are summarized in Table 12 for all films.

From the recorded spectra, no substantial changes were recorded after stress treatments. The main peaks were still present with a small change in the band intensity from increasing of the TDGS co-unit, associated with the increased chain mobility due to the presence of C–S bonds. A shift of no more than ±10 cm^−1^, as previously recorded by Siracusa et al. [30], was recorded with respect to the untreated samples. The shift was more evident after photoaging, as also seen for other properties such as molecular weight, thermal, mechanical, and gas barrier permeability. An increase of the –OH band intensity was recorded as evidence of the degradation process starting, and was more intense for samples with the highest TDGS co-unit content. These results were in agreement with the degradation study previously performed on such samples [11].

## 3. Materials and Methods

### 3.1. Synthesis of Poly(Butylene Succinate) Homopolymer and Poly(Butylene/Thiodiethylene Glycol Succinate) Copolymers

Polybuthylene succinate (PBS) and poly(butylene/thiodiethylene glycol succinate) P(BSxTDGSy) random copolymers were synthesized in bulk starting from dimethyl succinate (DMS), thiodiethylene glycol (TDG) and 1,4-butanediol (BD) and from different ratios of BD/TDG, using 20 mol % in excess of the total glycol content with respect to DMS. About 150 ppm of Ti(OBu)_4_/g of polymer was employed as a catalyst. Syntheses were carried out according to the experimental procedure previously reported by Genovese et al. [11], and the materials have been fully characterized from chemical–physical, mechanical, morphological, and permeability points of view.

### 3.2. Film Preparation and Thickness Determination

Films of PBS homopolymer and P(BSxTDGSy) copolymers were obtained by compression molding (Carver C12, Laboratory Press). Polymer powders were hot pressed between two Teflon sheets for 2 min at a temperature equal to T_m_ + 40 °C. The films were then cooled to room temperature in a press using running water. In order to attain equilibrium crystallinity, the films were stored at room temperature for at least two weeks prior to characterization.

The film thickness was determined using a digital dial indicator (Sample Thickness Tester DM-G, Unterhaching, Germany). The reported thickness represents the mean value of three experimental tests run in 10 different points on the polymer film surface at room temperature.

### 3.3. Thermal and Photoaging Procedures

The samples were exposed to thermal and photoaging, simulating the temperature aging process and supermarket exposure, respectively. Thermal aging was performed by a Constant Climate Chamber with Peltier Technology (model HPP 108/749, Memmert GmbH + Co. KG, P.O. Box 1720 D-91107, Schwabach, Germany) at 40 °C, using ventilated air. The relative humidity (RH) in the chambers was set at 50% RH, considered the average value recorded inside a supermarket within a solar year. These conditions correspond to an acceleration of real storage conditions, according to accelerated shelf life study recommendations [35,45,46].

The photoaging was carried out by exposing the polymer film samples to D65 Neon Light (the same used in a supermarket) at 23 °C and 50% of relative humidity. This light exposer is a homemade thermostat instrument, with a temperature and manual light controller. Samples were exposed from 0 to 55 days on a metal grid, at a distance of about 30 cm from the light. At selected time intervals, samples were collected in triplicate. Storage time was selected following the indications reported in the literature for a shelf life study on food [45,47].

### 3.4. Simulant Liquid

The food contact simulation was run in accordance with Regulation (EC) No. 1935/2004 of the European Parliament and of the council of 27 October 2004 on materials and articles intended to come into contact with food and in accordance with the Union Guidelines Regulation (*EU*) *No. 10/2011 on plastic materials and articles intended to come into contact with food* [48,49].

Four solutions were used as food simulants:

- Simulant A, Distilled Water, 10 days, 40 °C
- Simulant B, Acetic acid 3% (*v*/*v*), 10 days, 40 °C
- Simulant C, Ethanol 10% (*v*/*v*), 10 days, 40 °C
- Simulant D, Isooctane, 2 days, 20 °C

The measurements were made by soaking the material in the selected solutions (100 mL), in closed flasks. At the end of the test, specimens were removed from the flasks, washed with distilled water two times, and dried to a constant weight. Each material was tested in triplicate.

Test conditions resembled the worst foreseeable conditions of use regarding food contact time and temperature, as reported in Table 1, Table 2 and Table 3 of the EU Regulations [48,49]. As reported from the law, food simulants A–C are used for simulating the contact between materials and food that has a hydrolytic character and that are able to extract hydrophilic substances. Food simulant B is used for food that has a pH below 4.5, and food simulant C for alcoholic food with an alcohol content up to 20% and those foods that contain a relevant amount of organic ingredients that render the food more lipophilic. Food simulant D is used for foods that have a lipophilic character and are able to extract lipophilic substances and shall be used for alcoholic foods with an alcohol content of above 20% and for oil in water emulsions.

### 3.5. Permeability Measurement

The permeability determination was performed by a manometric method using a Permeance Testing Device, type GDP-C (Brugger Feinmechanik GmbH, München, Germany), according to ASTM 1434-82 (Standard test Method for Determining Gas Permeability Characteristics of Plastic Film and Sheeting), DIN 53 536 in compliance with ISO/DIS 15 105-1 and according to Gas Permeability Testing Manual (Registergericht München HRB 77020, Brugger Feinmechanik GmbH). The equipment consists of two chambers [37]. The upper chamber is filled with the dry test gases at ambient pressure. The permeation at the bottom chamber of the test specimen is determined by the evaluation of the pressure increase in the previously evacuated volume. Fluctuations of the ambient temperature during the test were controlled by software, with an automatic temperature compensation that minimizes Gas Transmission Rate (*GTR*) deviations.

Film samples of 2 × 2 cm in size were placed between the two chambers, using a film mask to cover the remaining surface area. The *GTR* (*cm*^3^/*cm*^2^ *day bar*)—that is, the Rate of Gas Transmission through the film—was determined considering the increase in pressure in relation to the time and volume of the device. The pressure is given by the instrument in (Bar) units. To obtain the data in kPa, the primary SI unit, it is necessary to use the following correction factor: 1 Bar = 100 kPa, according to NIST special publication 811 (NIST 2008) [50,51].

The time lag (*t_L_* in *seconds*), diffusion coefficient (*D*, in *cm*^2^/*sec*), and solubility (*S*, in *cm*^3^/*cm*^2^ *bar*) of the test gases into the film under study were measured in addition to *GTR*. The mathematical relations used are well reported in the literature [51,52,53].

Tests were carried out under the following operating conditions: 23 °C, with a relative humidity (RH) of 26%; gas stream of 100 cm^3^/min; 0% of Gas RH (Food Grade Gas); and sample area of 0.785 cm^2^.

Method A was used for the analysis, as reported in the literature [51,52,54], with evacuation of the top/bottom chambers.

Sample temperature was set by an external thermostat (KAAKE-Circulator DC10-K15 type, Thermoscientific, Selangor, Malaysia).

The transport phenomena background followed in the experiment is well described in literature, with a full description of the mathematical equation and interpretation [37,55].

All experiments were performed in triplicate and a good reproducibility was achieved. The mean value ± standard deviation is presented.

### 3.6. Thermal Analysis

Thermal transitions were measured by means of a Perkin Elmer DSC7 instrument (Waltham, MA, USA) equipped with a liquid sub ambient accessory and calibrated with high purity standards. Weighed samples of ca. 10 mg were encapsulated in aluminum pans and heated from −80 °C to about 40 °C above fusion temperature at a rate of 20 °C/min (first scan), held there for 3 min, rapidly quenched at 100 °C/min to −80 °C, and reheated to a temperature well above the melting point of the sample, at a heating rate of 20 °C/min (second scan). The melting temperature (T_m_) was determined as the peak value of the endothermal phenomena in the DSC curve. If multiple endotherms were observed, the highest peak was taken as T_m_. The heat of fusion (ΔH_m_) of the crystal phase was calculated from the total area of the DSC endotherm. At least three replicates were run for each sample.

### 3.7. Stress–Strain Measurements

Tensile testing was performed by using a Zwick Roell Texture machine mod Z2.5 (Zwick/Roell, Ulm, Germany), equipped with a rubber grip and 500 N load cell and controlled by a computer. A pre-load of 1 MPa and a speed of testing of 5 mm/min were applied. Rectangular films (5 mm wide and 50 mm high) were used. An initial grip separation of 20 mm and a crosshead speed of 5 mm/min were applied. All measurements have been carried out in accordance with ASTM D882-09.

All films were conditioned for 48 h at 23 °C and 50 ± 1% RH before testing, using a Constant Climate Chamber with Peltier Technology, model HPP 108/749.

At least six replicate specimens were run for each sample and the results were provided as the average value ± standard deviation.

### 3.8. Molecular Weight Determination

Molecular weight data were obtained by gel-permeation chromatography at 30 °C using the 1100 Hewlett Packard system (Palo Alto, CA, USA) equipped with PL gel 5 μ MiniMIX-C column and a refractive index detector. Chloroform was used as an eluent at a 0.3 mL/min flow rate and sample concentrations were of about 2 mg/mL. A molecular weight calibration curve was obtained by means of polystyrene standards in the molecular weight range 2000–1,000,000.

## 4. Conclusions

In the present work, a new class of biodegradable poly(butylene succinate) (PBS)-based random copolymers containing thioether-linkage of various compositions has been investigated and characterized for food packaging application. In particular, molecular, thermal, mechanical, and gas barrier properties have been evaluated after contact with four simulant liquids and after thermal and photoaging treatments. The results have been compared with the untreated starting materials. All the polymers showed a modification of the physical/chemical and mechanical properties after treatment.

As far as the mechanical properties are concerned, after contact with food simulant fluids, a general decrement of elastic modulus and elongation at break were found, with the exception of the richest sulfur atom copolymers, which showed an increased rigidity (higher elastic modulus). After thermal and photoaging, a worsening of mechanical properties was seen. This was found to be correlated with the copolymer composition: the higher the number of thioether linkages, the more significant the worsening. The sample with the highest TDGS percentage showed severe damage at the end of the experiment.

Regarding the permeability, PBS homopolymer was found to be characterized by different behavior with respect to its copolymers: gas barrier performance worsened after food simulant contact; worsened and then improved after thermal treatment, according to the exposure time; and kept constant after photo treatment. In the case of copolymers, the effects of treatment were more modest: after food simulant contact, no appreciable worsening of barrier performances was observed; after thermal and photo treatment, an improvement of barrier properties was found, well correlated with copolymer composition. The higher the number of TDGS co-units, the higher the improvement, due to inter-chain interactions, whose formation is favored by light and by the increasing amount of thioether linkages.

The sorption (thermodynamic parameter) and diffusion (kinetic parameter) processes are influenced by several factors such as polymer segments, intersegmental packing, chain flexibility, environment (polar or not), concentration of heteroatoms, and so on.

On the basis of the results obtained, the introduction of thioether linkages into the PBS backbone was revealed to be a powerful tool not only to tailor its material final properties in view of a desired application, but also to mitigate the effects due to thermal and photoaging. The presence of sulfur atoms even improves barrier performances after thermal and photo treatment, with the best effect being found after photo treatment.

It is worth remembering that polyesters are the polymers most extensively used for food packaging films. Production of biodegradable packaging films with good thermal and mechanical performance but the possibility of selecting the appropriate permeability could be a good alternative that would help solve the problem of food packaging polymer waste.

Furthermore, the study conducted simulating real use conditions allowed for a better understanding of how the chemical modifications due to different interaction with the environment could influence the physical, mechanical, and barrier properties of aliphatic polyester when used as food packaging.

## Figures and Tables

**Table 1:** Characterization data of the neat polymers (from [11]).

Polymer	M_n_	PDI	TDGS (mol %) by ^1^H NMR	WCA (°)	T_m_ (°C)	ΔH_m_ (J/mol)	Χ_c_ (%) by XRD	Thickness (μm)
PBS	51,000	2.7	0	90 ± 2	114	81	45	192 ± 4
P(BS80TDGS20)	54,000	2.8	20	83 ± 3	94	52	36	231 ± 20
P(BS60TDGS40)	51,000	2.8	36	80 ± 2	72	12	31	291 ± 7

PBS, poly (buthylene succinate); PTDGS, poly (thiodiethylene glycol succinate); PDI, polydispersity index; WCA, water contact angle.

**Table 2:** Residual M_n_ (%) after the treatment with food simulants.

Polymer	Simulant A	Simulant B	Simulant C	Simulant D
PBS	91	77	71	100
P(BS80TDGS20)	95	93	81	97
P(BS60TDGS40)	78	90	84	100

**Table 3:** Residual M_n_ (%) after thermal and photoaging in the range 0–55 days.

Polymer	Thermal	Photo	Thermal	Photo	Thermal	Photo
	10 Days		20 Days		55 Days	
PBS	86	79	85	70	82	63
P(BS80TDGS20)	87	79	84	76	82	60
P(BS60TDGS40)	80	56	78	36	73	n.d.

**Table 4:** Residual ΔH_m_ (%) after the treatment with food simulants.

Polymer	Simulant A	Simulant B	Simulant C	Simulant D
PBS	74	68	75	64
P(BS80TDGS20)	67	67	65	65
P(BS60TDGS40)	308	375	342	333

**Table 5:** Residual ΔH_m_ (%) after thermal and photoaging in the range 0–55 days.

Polymer	Thermal	Photo	Thermal	Photo	Thermal	Photo
	10 Days		20 Days		55 Days	
PBS	102	97	98	93	95	92
P(BS80TDGS20)	89	107	82	93	82	84
P(BS60TDGS40)	78	78	71	98	71	n.d.

**Table 6:** Mechanical characterization data of PBS and P(BSxTDGSy) copolymers, after treatment with food simulants.

Polymer	Untreated *		Simulant A		Simulant B		Simulant C		Simulant D	
	E (MPa)	ε_b_ (%)	E (MPa)	ε_b_ (%)	E (MPa)	ε_b_ (%)	E (MPa)	ε_b_ (%)	E (Ma)	ε_b_ (%)
PBS	440 ± 30	17 ± 2	431 ± 12	15 ± 2	428 ± 11	13 ± 1	413 ± 7	15 ± 2	378 ± 5	12 ± 1
P(BS80TDGS20)	260 ± 10	580 ± 70	213 ± 1	388 ± 15	251 ± 8	376 ± 14	265 ± 9	382 ± 17	264 ± 4	386 ± 7
P(BS60TDGS40)	160 ± 3	810 ± 20	178 ± 12	211 ± 17	181 ± 7	175 ± 15	173 ± 15	181 ± 13	188 ± 16	201 ± 4

* From [11,30].

**Table 7:** Mechanical characterization data of PBS and P(BSxTDGSy) copolymers after thermal aging in the range 0–55 days.

Polymer	Untreated *		Thermal		Thermal		Thermal	
	0 Days		10 Days		20 Days		55 Days	
	E (MPa)	ε_b_ (%)	E (MPa)	ε_b_ (%)	E (MPa)	ε_b_ (%)	E(MPa)	ε_b_ (%)
PBS	440 ± 30	17 ± 2	428 ± 22	16 ± 3	402 ± 12	14 ± 1	389 ± 21	14 ± 2
P(BS80TDGS20)	260 ± 10	580 ± 70	233 ± 27	567 ± 32	203 ± 15	556 ± 13	213 ± 11	560 ± 11
P(BS60TDGS40)	160 ± 3	810 ± 20	155 ± 8	802 ± 15	143 ± 1	789 ± 7	134 ± 2	713 ± 13

* From [11,30].

**Table 8:** Mechanical characterization data of PBS and P(BSxTDGSy) copolymers after photoaging in the range 0–55 days.

Polymer	Untreated *		Photo		Photo		Photo	
	0 Days		10 Days		20 Days		55 Days	
	E (MPa)	ε_b_ (%)	E (MPa)	ε_b_ (%)	E (MPa)	ε_b_ (%)	E (MPa)	ε_b_ (%)
PBS	440 ± 30	17 ± 2	418 ± 12	15 ± 1	402 ± 9	11 ± 1	378 ± 23	9 ± 2
P(BS80TDGS20)	260 ± 10	580 ± 70	241 ± 17	535 ± 16	178 ± 16	478 ± 9	165 ± 10	413 ± 19
P(BS60TDGS40)	160 ± 3	810 ± 20	133 ± 3	767 ± 23	111 ± 3	213 ± 7		

* From [11,30].

**Table 9:** *GTR*, *S*, *D*, and *t_L_* data for CO_2_ gas test, after food simulant contact *GTR* (*cm*^3^/*cm*^2^ *day bar*), *S* (*cm*^3^/*cm*^2^ *bar*), *D* (*cm*^2^/*sec*), *t_L_* (*sec*).

Permeability Parameters	Untreated *	Simulant A	Simulant B	Simulant C	Simulant D
*PBS*					
*GTR*	750 ± 1	914 ± 1 (>)	772 ± 2 (>)	909 ± 1 (>)	1023 ± 9 (>)
*S*	1.2 × 10^−1^ ± 0.1	1.1 ± 0.1 (>)	1.5 ± 0.0 (>)	6.4 ± 0.0 (>)	n.a.
*D*	1.4 × 10^−8^ ± 0.0	1.8 × 10^−8^ ± 0.0 (>)	8.4 × 10^−9^ ± 0.0 (<)	3.2 × 10^−8^ ± 0.0 (>)	n.a.
*t_L_*	4358 ± 529	3356 ± 279 (<)	5217 ± 16 (>)	1942 ± 10 (<)	n.a.
*P(BS80TDGS20)*					
*GTR*	1429 ± 2	1040 ± 0.0 (<)	990 ± 1 (<)	1460 ± 0 (>)	1860 ± 8 (>)
*S*	9.9 × 10^−1^ ± 0.0	1.4 ± 0.0 (>)	1.0 ± 0.1 (=)	1.0 ± 0.0 (=)	6.9 × 10^−1^ ± 0.0 (<)
*D*	3.9 × 10^−8^ ± 0.0	2.0 × 10^−8^ ± 0.0 (<)	2.6 × 10^−8^ ± 0.0 (<)	3.8 × 10^−8^ ± 0.0 (<)	7.3 × 10^−8^ ± 0.0 (<)
*t_L_*	2309 ± 122	4393 ± 17 (>)	3418 ± 207 (>)	2386 ± 5 (>)	1230 ± 30 (>)
*P(BS60TDGS40)*					
*GTR*	1722 ± 3	1573 ± 5 (<)	1596 ± 1 (<)	1178 ± 0 (<)	1243 ± 5 (<)
*S*	9.7 × 10^−1^ ± 0.1	1.0 ± 0.0 (>)	7.5 × 10^−1^ ± 0.1 (<)	1.4 ± 0.0 (>)	1.0 ± 0.0 (>)
*D*	6.1 × 10^−8^ ± 0.0	5.3 × 10^−8^ ± 0.0 (<)	7.2 × 10^−8^ ± 0.0 (>)	2.8 × 10^−8^ ± 0.0 (<)	4.1 × 10^−8^ ± 0.0 (<)
*t_L_*	6762 ± 130	2672 ± 4 (<)	1977 ± 214 (<)	4979 ± 15 (<)	3439 ± 11 (<)

* From [30].

**Table 10:** CO_2_/O_2_ ratios of permeability coefficients for untreated samples.

Polymer	*GTR* CO_2_/O_2_	*S* CO_2_/O_2_	*D* CO_2_/O_2_	*t_L_* CO_2_/O_2_
PBS	3.2	271.7	0.02	40.9
P(BS80TDGS20)	6.5	136.9	0.05	24.9
P(BS60TDGS40)	8.1	13.7	0.61	4.9

**Table 11:** R^2^ value for PBS and P(BSxTDGSy) copolymers after thermal aging and photoaging.

Polymer	Thermal	Photo	Thermal	Photo	Thermal	Photo	Thermal	Photo
	*GTR*	*GTR*	*S*	*S*	*D*	*D*	*t_L_*	*t_L_*
PBS	0.036	0.060	0.164	0.167	0.043	0.471	0.058	0.456
P(BS80TDGS20)	0.546	0.338	0.006	0.027	0.243	0.065	0.217	0.469
P(BS60TDGS40)	0.038	0.347	0.009	0.378	0.001	0.032	0.004	0.097

**Table 12:** FT-IR data for PBS and P(BSxTDGSy) films.

Chemical Group	Peak Position, cm^−1^
–OH stretch (free)	3571
CH-stretch (of CH_2_)	2918 (ν_as_ CH_2_), 2856 (ν_s_ CH_2_)
–C=O normal carbonyl stretch	1712
–CH-deformation symmetric and asymmetric bending	1472 (δ_s_ CH_2_)
C-O-H in-plane bend	1424
–CH_2_-scissoring	1387
–C=O bending	1245
–C-O stretching	1178, 1153
–OH bending	1046
–CH_2_ wagging and twisting	1244, 1178
–CH_2_ rocking	751
O-H out-of-plane	994 (as), 955(s)
C-C stretch	920, 809
C-S stretch	743

From [30].
